# Assessing the generalisability of radiomics features previously identified as predictive of radiation-induced sticky saliva and xerostomia

**DOI:** 10.1016/j.phro.2022.12.001

**Published:** 2022-12-16

**Authors:** Thomas Berger, David J. Noble, Zhuolin Yang, Leila E.A. Shelley, Thomas McMullan, Amy Bates, Simon Thomas, Linda J. Carruthers, George Beckett, Aileen Duffton, Claire Paterson, Raj Jena, Duncan B. McLaren, Neil G. Burnet, William H. Nailon

**Affiliations:** a Department of Oncology Physics, Edinburgh Cancer Centre, Western General Hospital, Crewe Road South, Edinburgh EH4 2XU, UK; b Edinburgh Cancer Research Centre, Institute of Genetics and Cancer, The University of Edinburgh, Edinburgh, UK; c The University of Cambridge, Department of Oncology, Cambridge Biomedical Campus, Hills Road, Cambridge CB2 0QQ, UK; d Department of Clinical Oncology, Edinburgh Cancer Centre, Western General Hospital, Crewe Road South, Edinburgh EH4 2XU, UK; e School of Engineering, the University of Edinburgh, the King’s Buildings, Mayfield Road, Edinburgh EH9 3JL, UK; f Department of Medical Physics and Clinical Engineering, Cambridge University Hospitals NHS Foundation Trust, Cambridge Biomedical Campus, Hills Road, Cambridge CB2 0QQ, UK; g Edinburgh Parallel Computing Centre, Bayes Centre, 47 Potterrow, Edinburgh EH8 9BT, UK; h Beatson West of Scotland Cancer Centre, Great Western Road, Glasgow G12 0YN, UK; i The Christie NHS Foundation Trust, Wilmslow Road, Manchester M20 4BX, UK

**Keywords:** Radiomics, Replication, Xerostomia, Sticky saliva, Head and neck cancer, Image analysis

## Abstract

**Background and purpose:**

While core to the scientific approach, reproducibility of experimental results is challenging in radiomics studies. A recent publication identified radiomics features that are predictive of late irradiation-induced toxicity in head and neck cancer (HNC) patients. In this study, we assessed the generalisability of these findings.

**Materials and Methods:**

The procedure described in the publication in question was applied to a cohort of 109 HNC patients treated with 50–70 Gy in 20–35 fractions using helical radiotherapy although there were inherent differences between the two patient populations and methodologies. On each slice of the planning CT with delineated parotid and submandibular glands, the imaging features that were previously identified as predictive of moderate-to-severe xerostomia and sticky saliva 12 months post radiotherapy (Xer12m and SS12m) were calculated. Specifically, Short Run Emphasis (SRE) and maximum CT intensity (maxHU) were evaluated for improvement in prediction of Xer12m and SS12m respectively, compared to models solely using baseline toxicity and mean dose to the salivary glands.

**Results:**

None of the associations previously identified as statistically significant and involving radiomics features in univariate or multivariate models could be reproduced on our cohort.

**Conclusion:**

The discrepancies observed between the results of the two studies delineate limits to the generalisability of the previously reported findings. This may be explained by the differences in the approaches, in particular the imaging characteristics and subsequent methodological implementation. This highlights the importance of external validation, high quality reporting guidelines and standardisation protocols to ensure generalisability, replication and ultimately clinical implementation.

## 1 Introduction

Research in the area of *radiomics* has developed rapidly over the last decade. Since the introduction of the term in 2010 [1], [2], the number of results on PubMed pertaining to this keyword increased from 3 in 2012 to 2269 in 2021. Until now the majority of articles have been investigative studies [3] reporting on the development of predictive models using datasets originating from a single centre [4], [5], [6]. However the discipline of radiomics has matured at a fast pace, with substantial evolution in publication standards in a relatively short time. Radiomics is often presented as a potential solution to better personalised care which will eventually result in improved clinical outcomes. However, to date, and despite the soaring number of investigative studies, prospective clinical trials have yet to demonstrate the full prognostic or predictive benefit of a radiomics signature in radiation oncology. Very few of these investigative studies will ever lead to a direct benefit for cancer patients, as very few include external validation, and replication studies remain rare and complex. While core to the scientific approach, reproducibility of experimental results is often a challenge for radiomics studies because of the complexity of the methods.

Indeed, there are many methodological steps involved in radiomics analyses, including image acquisition, organ contouring, image processing, statistical analysis, incorporation of clinical outcome scales, with each requiring the adjustment of a multitude of parameters. It is therefore extremely challenging to have materials from different cohorts as well as subsequent analysis steps with sufficiently comparable demographic, clinical and technical characteristics to qualify for a complete replication study.

To partially address these difficulties, several initiatives have been proposed to harmonise some of the aforementioned methodological steps. In particular, the Image Biomarker Standardisation Initiative (IBSI) [7], [8], COMBAT (combating batch effects when combining batches) [9], [10], [11], [12], [13] and the Transparent Reporting of a multivariable prediction model for Individual Prognosis Or Diagnosis (TRIPOD) [14], [15] provide a framework for the standardisation of radiomics calculation, data acquired on different machines and the reporting of the methods. Despite the potential improvements from the adoption of these initiatives across the community, it remains almost impossible to remove differences in all the links of the methodological chain spanning image acquisition to outcome prediction. In a complementary approach, researchers have investigated the repeatability and reproducibility of radiomics features and found them to be sensitive at various levels to some of the previously described parameters [16].

In this context, assessing the generalisability of previously identified radiomics signatures by evaluating their predictive performance on new cohorts addresses an important scientific matter where there is a significant gap in the current literature.

Several investigative studies have shown how imaging characteristics from varying modalities predict clinical outcomes of interest [17], [18], [19], [20], [21], [22]. A sub-set of these have demonstrated that radiomics features extracted from the salivary glands predict radiation-induced salivary dysfunction in patients with cancer in the head and neck region (HNC) [23], [24], [25], [26], [27], [28]. In one such study, van Dijk et al reported that specific radiomics features extracted on pre-treatment planning CTs improved prediction of moderate-to-severe (EORTC-QLQ-HN35 G ≥ 3) sticky saliva (SS12m) and xerostomia (Xer12m) at 12 months after radiotherapy. The gain in predictive performance which was found to be modest but statistically significant was evaluated in comparison with models based only on dose and clinical parameters.

The aim of the present study is to assess the generalisability of the findings of van Dijk et al by applying their methodology, to the best of our ability, to another cohort of HNC patients also treated with radiotherapy.

## 2 Materials and methods

### 2.1 Demographic and clinical characteristics

The present study consisted in the analysis of the data of 109 HNC patients collected as part of the UK Clinical Research Network (UKCRNID:13716) VoxTox study [29], [30] which received approval from the National Research Ethics Service Committee East of England (13/EE/0008) in 2013. All patients were treated in Cambridge using TomoTherapy HiArt machines (Accuray, Sunnyvale, CA, USA) with 50–70 Gy delivered in 20–35 fractions. Using the same EORTC QLQ-HN35 questionnaire as in van Dijk et al’s study, xerostomia and sticky saliva scores were prospectively collected at baseline and 12 months after radiotherapy treatment. The endpoints of interest of the present study were Xer12m and SS12m, as defined in van Dijk et al’s work and corresponding to grades ≥ 3 on the EORTC scale. The demographic and clinical characteristics of the patients analysed are shown in detail and compared to van Dijk et al’s cohort in Table 1. Patients with primary tumours in the salivary glands were excluded to proceed identically to van Dijk et al. However, conversely to their approach, exclusion of patients with excised parotid or submandibular glands (PG & SMG) could not be performed because it would have resulted in a sub-optimal sample size. Those with a contra-lateral parotid and, for sticky saliva prediction, at least one sub-mandibular gland were therefore included.

**Table 1:** Patients demographic and treatment characteristics.

		van Dijk et al	This study
	Number of patients	249	109
	Contra-lateral parotid mean dose median [Q1–Q3] (Gy)	Not found	29.2 [14.6–34.9]
	Bilateral submandibular mean dose median [Q1–Q3] (Gy)	Not found	56.4 [47.2–59.5]

Moderate-to-severe xerostomia	Baseline	Not found	7 (6 %)
	12 months	100 (40 %)	52 (48 %)

Moderate-to-severe sticky saliva	Baseline	Not found	7 (6 %)
	12 months	63 (25 %)	36 (33 %)

Age	Median [Q1–Q3] (years)	Not found	59 [53–65]
	18–65 years	133 (53 %)	84 (77 %)
	>65 years	116 (47 %)	25 (23 %)

Disease primary site	Oropharynx	74 (30 %)	71 (65 %)
	Oral cavity	11 (4 %)	8 (7 %)
	Unknown primary	1 (0 %)	7 (6 %)
	Skin	0	6 (6 %)
	Larynx	118 (47 %)	6 (6 %)
	Maxilla	0	5 (5 %)
	Nasopharynx	14 (6 %)	3 (3 %)
	Hypopharynx	31 (12 %)	2 (2 %)
	Carotid body	0	1 (1 %)

Dose prescription	50 Gy	0	1 (1 %)
	55 Gy	0	1 (1 %)
	60 Gy	0	24 (22 %)
	65 Gy	0	80 (73 %)
	70 Gy	249 (100 %)	3 (3 %)

Irradiation technique	3D-CRT	23 (9 %)	0
	Fixed field IMRT	216 (87 %)	0
	Volumetric arc therapy	10 (4 %)	109 (100 %)

Bilateral irradiation	None	46 (18 %)	31 (28 %)
	Yes	203 (82 %)	78 (72 %)

Systemic treatment	None	149 (60 %)	36 (33 %)
	Yes	100 (40 %)	73 (67 %)

tumour stage TNM (AJCC) 7	T0-2	111 (45 %)	73 (67 %)
	T3-4	138 (55 %)	36 (33 %)

Nodal status TNM (AJCC) 7	N0-1	138 (55 %)	40 (37 %)
	N2-3	111 (45 %)	69 (63 %)

The radiation protocols used to treat this cohort are described in detail in Noble et al. [31] and while broadly similar to those utilised by van Dijk et al. [32], they differ in some aspects as summarised in Table 1.

The mean doses to both the contra- and bi-lateral PGs and SMGs were determined and later used, in an approach identical to van Dijk et al, in multi-variate logistic regression models to predict Xer12m and SS12m, respectively.

A software suite was developed in-house using MatLab (MathWorks, MA, USA) for analysing images while the dose analysis was performed using Computational Environment for Radiological Research (CERR) [33], [34].

### 2.2 Imaging characteristics

PGs and SMGs were delineated on pre-treatment planning CTs (Toshiba Aquilion/LB, 120 kV, voxel size: 1.074 × 1.074 × 3.0 mm^3^) according to the same contouring protocols as van Dijk et al. [35], [36].

To avoid analysing CT intensity values that do not correspond to tissue densities [37], [38], van Dijk et al excluded 33 % of their patients as they presented with metal artefacts on their CT scans. Implementing the same approach in our cohort, would have resulted in the exclusion of 95 % (104/109) of the patients and was therefore not undertaken. This difference is remarkable and the proportion of patients with dental implants in our cohort is similar to the one reported by the NIH National Institute of Dental and Craniofacial Research with 92 % of adults who have had dental caries in their permanent teeth [39].

### 2.3 Radiomics features extraction

In the study conducted by van Dijk et al, a total number of 130 CT imaging features composed of first order statistics, shape- and size-based as well as textural features were considered to build their predictive models. In the present work the only radiomics features extracted were those identified by van Dijk et al from the 130 available as being statistically significantly associated with the endpoints of interest. For univariate associations, we considered the radiomics features presented in Table 2 of their publication [28] which are derived from first order statistics features, Gray Level Co-occurrence Matrix (GLCM) and Gray Level Run-Length Matrix (GLRLM). With regards to multi-variate logistic regression models, they found that the addition of Short Run Emphasis (SRE), a GLRLM-derived feature, and maximum CT intensity (maxHU) improved prediction of Xer12m and SS12m respectively, compared to logistic regression models only using baseline toxicity scores and mean dose to the salivary glands.

**Table 2:** Summary of the major similarities and differences between the approach of van Dijk et al and our study.

	Category		Van Dijk et al	This study
Differences	Radiomics features	Texture features	3D (13 directions)	2D (4 directions)
		Standardization protocol	Not specified	Image Biomarker Standardization Initiative
	Dose and fractionation	Tumour region	70 Gy in 35fx	50–70 Gy in 20-35fx (95 % had 30fx)
	Exclusion criteria	Dental implants	Patients excluded	CT slices excluded
		Resected salivary glands	Patients excluded	Tolerance on salivary gland resection
	CT images	Manufacturer	Siemens	Toshiba
		Model	Somatom Sensation Open	Aquilion/LB
		Voxel size (mm^3^)	0.94 × 0.94 × 2.0	1.074x1.074x3
		Energy	100–140 kV	120kVp
		contrast agent pre-acquisition	Yes	76 % (following department protocol)
	Disease primary site (detailed in Table 1)		47 % larynx, 30 % oropharynx, 12 % hypopharynx…	65 % oropharynx, 7 % oral cavity, 6 % unknown primary…
	Treatment Technique (detailed in Table 1)		3D-CRT/IMRT/VMAT	TomoTherapy

Similarities	First order statistics features		3D	3D
	Contouring protocol		Brouwer et al.	Grégoire et al. & Brouwer et al.
	Toxicity scoring		EORTC QLQ-HN35	EORTC QLQ-HN35
	Number of patients		249	109

The radiomics features calculated in this analysis are IBSI-compliant [8] and were benchmarked using resources from the IBSI website [40]. The formula used were identical or equivalent to the ones van Dijk et al utilised, judging by the details provided in the paper they referenced [41]. The feature 'GLCM homogeneity squared' in van Dijk et al’s paper appears to be equivalent to the feature ‘homogeneity 2′ in the Aerts et al publication [41] which corresponds to the 'inverse difference moment' according to the IBSI terminology, which is the term we chose to use here.

As we excluded CT slices with dental implants and because textural features, in particular GLRLM and GLCM, are sensitive to the position of voxels relatively to one another, it is not sensible to calculate these in the superior-inferior directions because the exclusion of CT slices results in disjointed runs of voxels in this axis. Therefore, GLCM and GLRLM features were calculated on each selected transversal slice of the relevant salivary gland contour in four directions and then averaged using the aggregation method referred to as BTW3 in the IBSI. In this respect, our approach is deviating from van Dijk et al’s, as they reported to have calculated their texture matrices considering 26-connected voxels in 13 directions in three dimensions.

Prior to implementing textural feature calculations, and in an identical manner as van Dijk et al, voxel intensities were resampled into 25-Hounsfield-Unit bins from −200 HU to 200 HU. Also, features were normalised by subtracting the mean and dividing by the standard deviation across patients.

### 2.4 Statistical analysis

In the present study, we evaluated the predictive performance of the radiomics features identified by van Dijk et al as being predictive of the outcomes of interest and presented in their Table 2. Therefore, no feature selection was needed and to benefit from the greatest statistical power, the predictive performance was assessed on the whole data set.

For statistical analyses, R [42] and the ROCit [43] and lmtest packages [44] were utilised. The test used by van Dijk et al to compute p-values for univariate associations was not found explicitly mentioned and we therefore chose to use a p-value computed by the glm function in univariate associations. The models’ performance was assessed by using the Area Under the Curve (AUC) metric. Also, in a way similar to van Dijk et al we performed likelihood ratio tests to determine whether the added value of the radiomics feature is statistically significant compared to the models composed only of mean dose and baseline toxicity score.

### 2.5 Influence of the differences between the two approaches on predictive performance

The differences between the two approaches are summarised in Table 2. To investigate whether these differences impacted model performance, tests were run on subgroups of patients with each group having a varying proportion of patients with: 1) intact salivary glands, 2) excluded CT slices with dental implants, and 3) consistent fractionation schedules.

In a separate approach, 3D calculation of the SRE feature was implemented for the PGs of the five patients that did not present with dental implants and compared, by the means of the Pearson correlation coefficient, with results obtained in 2D.

## 3 Results

Of the 109 patients analysed in this study, 52 (48 %) and 36 (33 %) reported symptoms of Xer12m and SS12m, respectively.

As shown in Table 3, the only predictor tested that was found to be statistically significantly associated with the outcomes of interest is the mean dose to the relevant salivary glands with a p-value of 0.043 and 0.012 for Xer12m and SS12m prediction, respectively. None of the univariate associations involving radiomic features identified by van Dijk et al as being statistically significant could be reproduced on our cohort (Table 3).

**Table 3:** Predictive performance of the predictors identified by van Dijk et al on the different groups of patients analysed.

Histograms showing the distribution of patients with intact salivary glands, excluded CT slices due to dental implants, and consistent fractionation schedules are displayed in Supplementary Fig. 1. Of the 109 patients with at least one contra-lateral PG, 105 had both intact parotids and 106 had an SMG intact. In total, 84 patients had both SMGs intact and 80 had all four of their major salivary glands. The presence of metal implants led to the exclusion of < 50 % of the CT slices of the contra-lateral PG in 87 patients. With regards to the fractionation schedule, 80 patients were treated with 65 Gy delivered in 30 fractions.

**Fig. 1:**
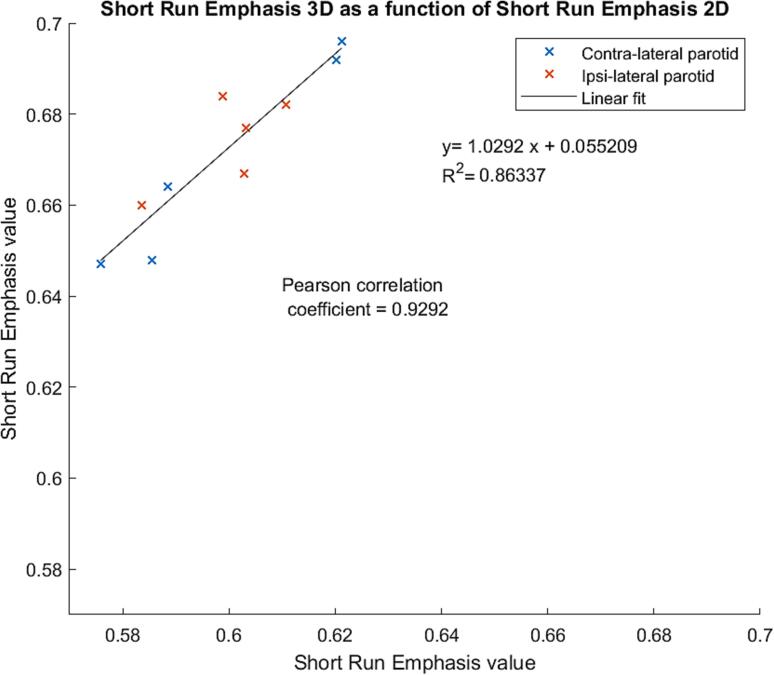
Relationship between 3D calculation of the short run emphasis feature with results obtained in 2D for the parotid glands of the five patients that did not present with dental implants.

As illustrated in Fig. 1, for the subsample of PGs analysed, the SRE values calculated in 2D are highly correlated with those calculated in 3D with a Pearson correlation coefficient of 0.93. However, the rankings of the parotids are not identical with both methods.

The addition of SRE to the standard model did not significantly improve Xer12m prediction (p > 0.78) on any of the subgroups of patients tested. For patients with both SMGs intact, the addition of the feature maxHU increased the AUC from 0.53 to 0.66 but this difference is not considered statistically significant (p = 0.50) and the AUCs remain markedly smaller than the ones found by van Dijk et al (0.74 and 0.77).

## 4 Discussion

In the cohort analysed, none of the radiomics-based univariate associations identified by van Dijk et al as statistically significant could be reproduced. The addition of SRE to the standard model did not improve significantly Xer12m prediction on any of the subgroups tested. While the addition of maxHU improved SS12m prediction on patients with both intact SMGs, this gain is not statistically significant and the AUCs remain markedly smaller than those found by van Dijk et al. These discrepancies, which limit the generalisability of van Dijk et al’s findings, may be explained by the differences between the two studies and in particular in the imaging characteristics and methodological implementation.

In the present study, textural features were calculated on 2D transversal slices because of the presence of dental implants for a large proportion of patients which necessitated the exclusion of some slices. When analysing the subsample of parotids without metal artefacts, 2D and 3D features were found to be highly correlated, but the ranking of the parotids was found not to be identical, which may impact the predictive performance to some extent. This is a major difference with van Dijk et al’s approach, as all their feature values and in particular those derived from GLCM and GLRLM matrices were calculated in 3D. Also, while the first-order-statistics features’ formulas were identical in both studies, such as for maxHU used in the multi-variate model predicting SS12m, the exclusion of some CT slices in our approach may still result in discrepancies.

The two approaches also deviate in other points that are listed in Table 1, Table 2. In particular, it is interesting to note that the proportion of patients with systemic treatment administered differs widely between the two cohorts (67 % in this cohort against 40 % in van Dijk et al’s). Systemic treatment is overall a known factor of toxicity development and may thus interfere with the relevance of a radiomics signature. Also, the distributions of patients in the two cohorts differ significantly in age and in primary cancer sites. Patients treated for a primary tumour located in the oropharynx, which represent the largest group in our cohort (with 65 % of patients against 30 %) can be expected to have on average higher parotid doses than those treated for a tumour in the larynx, which is the predominant indication in van Dijk et al’s cohort (47 % of patients).

However, the differences in the materials’ characteristics which could be studied using subgroups of patients did not have a significant impact on the predictive performance of the models. Another important aspect to be considered here is the number of patients included in the analyses and the statistical power associated, as van Dijk et al found modest increases in predictive performance with an AUC increase from 0.75 to 0.77 and 0.74 to 0.77 for xerostomia and sticky saliva prediction in a cohort composed of 249 patients. The 109 patients included in the present study may therefore not be sufficient to reveal the statistical significance of the added value of these imaging features. This rationale was also used by van Dijk et al to explain, in another study they conducted (which investigated ^18^F-FDG PET image biomarkers for xerostomia prediction), that the addition of SRE calculated on planning CTs on a subset of 100 patients was found not to significantly improve the predictive performance compared to a reference model [23].

Another aspect which may explain the differences in predictive performance is that it appears that van Dijk et al performed their feature selection on the whole dataset, which has been shown as potentially leading to overfitting/optimism [45].

In their study on CT biomarkers, van Dijk et al interpret that the predictive power of SRE may be explained by the fact it reflects parotid tissue heterogeneity which is associated with adipocyte presence and a lower proportion of functioning parenchyma within the gland. This theory is corroborated by their complementary studies in which they used different imaging modalities [23], [26]. In a letter to the Editor, Nardone et al also announced preliminary results that seem to corroborate these findings [46].

It is highly improbable for two distinct medical patients’ datasets to have all acquisition and analysis parameters/characteristics identical. The almost inevitable presence of a differing link in the whole analysis chain, results in an unrealisable testing of refutability of radiomics results, as the cause of the discrepancy in predictive power can always sensibly be thought to originate from one of the differing links. This difficulty or impossibility to perfectly replicate a study and hence to refute radiomics findings may in turn hamper the progression of scientific knowledge as researchers are then constrained to assess the generalisability of radiomics models. This is compounded further by a growing number of theories in this area facilitated by the acceleration of our discipline [47], which cannot be empirically regulated.

To conclude, the findings of this study highlight the challenges of generalisability of radiomics features previously identified as predictive of radiation-induced sticky saliva and xerostomia. In particular, none of the statistically significant associations involving radiomics features identified by van Dijk et al in univariate or multivariate models could be reproduced on our cohort of HNC patients. These variations may be explained by the numerous differences present between the two studies. In particular here, imaging characteristics and subsequent methodological implementation differed. Similar divergences will inherently be present to some extent when applying a model from the literature under realistic clinical conditions. However, this should not deter further investigation on radiomics, such as in this study, as this may contribute to guide the design of future clinical trials or standardisation initiatives. The limited generalisability observed here highlights the importance of external validation and in particular the need for high quality reporting guidelines and standardisation protocols to ensure generalisability, replication and ultimately clinical implementation.

## Declaration of Competing Interest

The authors declare the following financial interests/personal relationships which may be considered as potential competing interests: NB, LJC, RJ, AB, WHN, DJN, GB, LEAS, TB, DM, TM report grants from Chief Scientist Office (CSO) Scotland grant (TCS/17/26 - CSO Award), during the conduct of the study. The authors alone are responsible for the content and writing of the paper. LJC reports personal fees from BrainLAB - Novalis Certified, outside the submitted work; RJ reports personal fees from Microsoft, outside the submitted work; DJN reports grants from Cancer Research UK Clinical Research Fellowship (Ref: C20/A20917), grants from Cancer Research UK Programme Grant (Ref: C8857/A13405), during the conduct of the study; LEAS reports grants from University of Cambridge WD Armstrong Trust, outside the submitted work. AD, CP, and ST have nothing to disclose.
