# Supplementary material for: Assessing the generalisability of radiomics features previously identified as predictive of radiation-induced sticky saliva and xerostomia
Source: Phys Imaging Radiat Oncol. 2022 Dec 16;25:100404. doi: 10.1016/j.phro.2022.12.001 (PMC9843480; doi:10.1016/j.phro.2022.12.001)

Supplementary Figure 1

Histograms showing the distribution of patients with intact salivary glands, excluded CT slices due to dental implants, and consistent fractionation schedules

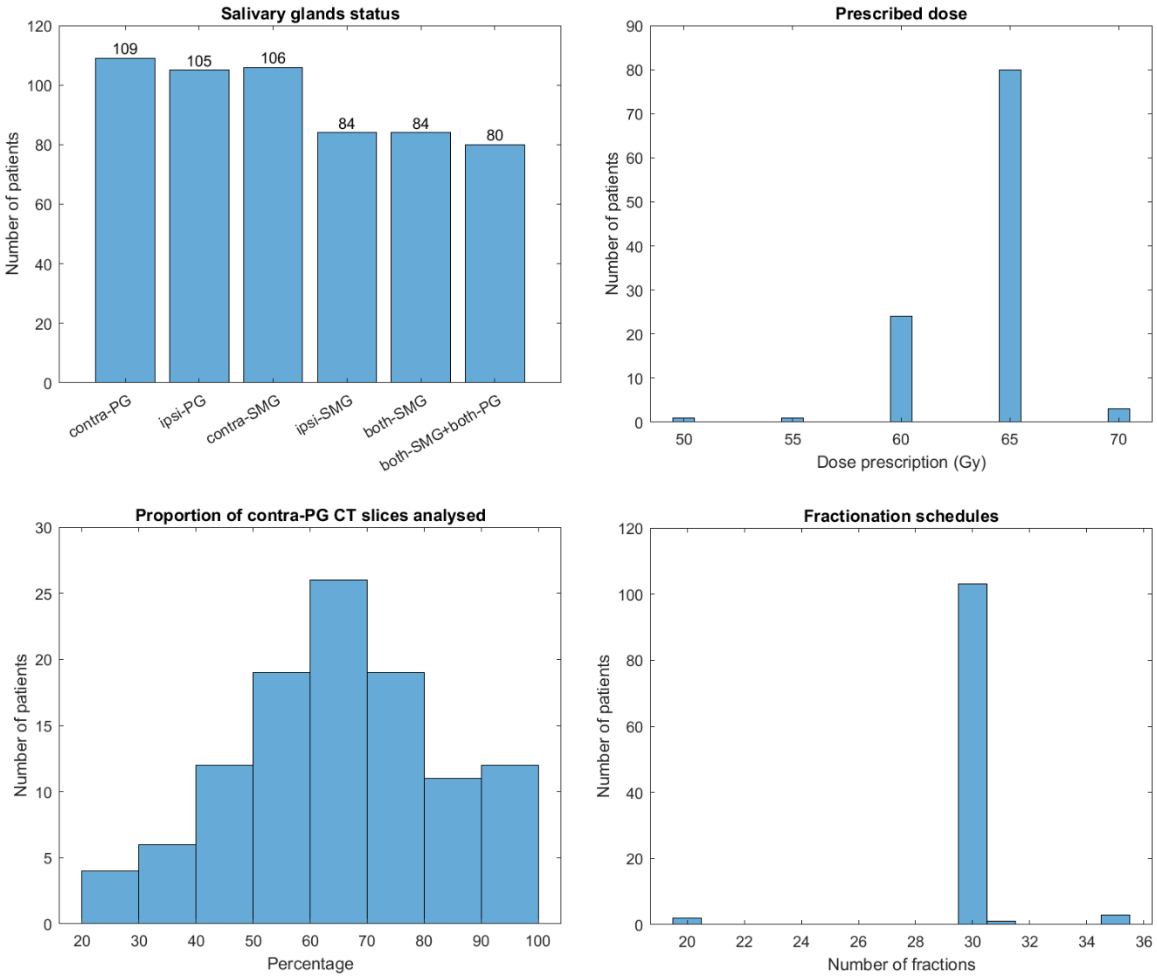

Supplement: Supplementary data 1 [file mmc1.pdf]
